# Comprehensive profiling of host- and virus-derived circular RNAs during vesicular stomatitis virus infection

**DOI:** 10.3389/fcimb.2025.1654185

**Published:** 2025-10-16

**Authors:** Shanshan Miao, Zesen Mai, Lu Zhu, Mingzhen Lin, Xinru Yang, Yezhenghong Qiu, Yi Wang, Zhaoyu Liu, Wenxia Yao

**Affiliations:** ^1^ Key Laboratory of Biological Targeting Diagnosis, Therapy and Rehabilitation of Guangdong Higher Education Institutes, GuangDong Engineering Technology Research Center of Biological Targeting Diagnosis, Therapy and Rehabilitation, State Key Laboratory of Respiratory Disease, The Fifth Affiliated Hospital, Guangzhou Medical University, The Fifth Clinical College of Guangzhou Medical University, Guangzhou, China; ^2^ Department of Dermatology, The Fifth Affiliated Hospital, Guangzhou Medical University, The Fifth Clinical College of Guangzhou Medical University, Guangzhou, China; ^3^ Dongguan Key Laboratory for Zoonitic Diseases, Dongguan Bioshine Technology Company, Dongguan, China

**Keywords:** vesicular stomatitis virus1, RNA-seq2, cellular circRNAs3, viral circRNAs4, differentially expressed circRNAs5

## Abstract

Circular RNA (circRNA) is a new member of noncoding RNA family, which has garnered increasing attention, particularly in the context of viral infections. Vesicular stomatitis virus (VSV) is a negative-sense RNA virus that threatens animal husbandry and currently lacks effective treatments. Despite extensive studies on VSV in basic research and medical applications, the systemic profiling of circRNAs in the context of VSV remains unexplored. In this study, we conducted a comprehensive analysis of circRNA profiles in VSV-infected Vero cells using high-throughput sequencing. We identified a total of 65,645 host-derived cellular circRNAs, of which 1,682 were differentially expressed. Trend clustering revealed three significant expression patterns, and functional annotation indicated that cluster 1 was associated with proviral pathways. Subsequent results showed that VSV infection elevated the top 10 cellular circRNAs, which in turn promoted VSV replication. Additionally, we identified 120 virus-derived circRNAs, top 10 of which were upregulated by VSV and enhanced VSV infection as well. We also characterized the general features of both cellular and viral circRNAs, including genomic locations and back-splicing signals. In summary, our findings revealed that both host cellular and viral circRNAs are induced by VSV infection, subsequently affecting VSV infection. This study unveils a previously unrecognized layer of virus-host interactions involving circRNAs, which may assist in the development of control strategies for VSV and its fundamental and medical applications.

## 1 Introduction

Vesicular stomatitis virus (VSV), a non-segmented negative-sense RNA virus in the genus *Vesiculovirus* (Rhabdoviridae), possesses an 11.2 kb genome encoding five genes (N-P-M-G-L) that serve as templates for mRNA and anti-genome synthesis (Dietzgen et al., 2017). Traditionally, VSV has been regarded as an important veterinary pathogen due to its high pathogenicity to domestic animals, including cattle, horses, and swine. The infection causes vesicular stomatitis, characterized by painful lesions in domestic animals, including cattle, horses, and swine (Letchworth et al., 1999), and morbidity rates can vary widely, reaching high up to 90% in some herds, which can cause significant economic losses and cross-border epidemics. In humans, VSV infection can cause flu-like symptoms and rarely oral lesions (Pelzel-McCluskey et al., 2021). Although the infections are mostly self-limiting in humans, the zoonotic potential of VSV highlights its relevance to both animal health and public health under the ‘One Health’ framework. Their transmission remains a threat to animal husbandry and public health. Currently, no vaccine is available to prevent VSV. With growing research, VSV has expanded beyond veterinary virology and now provides unique opportunities in both fundamental science and medical applications. The simple genomic structure and flexible genomic plasticity make VSV an ideal model for studying RNA virus evolution; its efficient replication, and infecting a wide range of mammalian cells facilitates research into virus-host interactions; and its efficient evasion of host defenses—such as suppressing innate immune signaling and modulating apoptosis and autophagy—provides a powerful system for dissecting viral immune escape mechanisms (Stojdl et al., 2003; Hastie and Grdzelishvili, 2012; Ahmed et al., 2024). Despite its compact genome, recent long-read sequencing (LRS) studies have revealed architectural complexity in VSV transcripts (Wongsurawat et al., 2019; Kakuk et al., 2021), highlighting the potential for further novel RNA species to be identified. In addition, VSV has emerged as a promising platform for therapeutic medicine including vaccine development and oncolytic applications in recent years (Ahmed et al., 2024), paving the way for improved disease prevention and control strategies in both animals and humans. A notable success is the VSV-based Ebola virus vaccine, rVSV-ZEBOV, which was used during the outbreak and demonstrated encouraging protective efficacy (Regules et al., 2017). Moreover, a prominent advancement in oncolytic therapy is the engineered VSV-IFNβ-NIS, which has demonstrated favorable tumor selectivity and safety and induced antitumor efficacy in clinical trials (McGarrah et al., 2023; Ahmed et al., 2024).A deeper understanding of VSV replication in host cells is crucial for developing prevention and control strategies for VSV, as well as advancing its fundamental and medical applications.

In recent years, with rapid advancements in non-coding RNA research, circular RNAs (circRNAs) have emerged as a novel regulatory layer, attracting considerable attention. CircRNAs are characterized by tissue-specific expression patterns and lack of 5’ caps and 3’ poly(A) tails, which confer resistance to exonucleases. An increasing number of studies have revealed that circRNAs play roles in various physiological and pathological processes, as well as in disease development. These roles have been linked to mechanisms such as modulating transcription and splicing, regulating the stability and translation of cytoplasmic mRNAs, interfering with signaling pathways, and even serving as templates for translation (Liu and Chen, 2022). This functional diversity, combined with their specific expression and stability, has made circRNA an increasingly prominent research focus. In the field of viral-host cell interactions, circRNAs have recently elicited increased attention (Tan and Lim, 2021). On the one hand, host viral infection induces a host cell’s circRNA expression profile, which is further utilized by the virus or host cell (Choudhary et al., 2021). For instance, Kaposi’s sarcoma-associated herpesvirus (KSHV) infection induces the upregulation of the host circRNA hsa_circ_0001400, which suppresses viral gene expression (Tagawa et al., 2018). On the other hand, studies regarding circRNAs derived from viruses and their roles are continuously emerging. In earlier years, research teams discovered and validated the presence of viral circRNAs, primarily in DNA viruses (Toptan et al., 2018; Ungerleider et al., 2018). Later, our research, alongside studies from other teams, found that circRNAs can also be derived from certain RNA viruses, including respiratory syncytial virus, hepatitis C virus, and betacoronaviruses (Yao et al., 2021; Yang et al., 2022; Cao et al., 2024). These circRNAs can serve as potential biomarkers for the diagnosis and prognosis of virus infection (Wang et al., 2024), and various biological roles have been ascribed to viral circRNAs, including encoding oncoproteins and regulating viral replication (Zhao et al., 2019; Zhang et al., 2022). For example, a recent study identified a functional circRNA encoded by the RNA virus Bombyx mori Nucleopolyhedrovirus, which translates a viral small peptide VSP39, that promotes viral replication (Zhang et al., 2023). Therefore, viral circRNAs could serve as diagnostic and therapeutic targets, offering a foundation for the prevention and diagnosis of viral diseases.

Although studies on circRNAs and their roles in viral infections continue to emerge, they remain insufficient. Further research is required to gain a better understanding of viral-host interactions in the layer of circRNAs, particularly for RNA viruses. VSV, an RNA virus lacking effective treatments or vaccines, has been extensively studied for its fundamental and therapeutic applications. However, the systemic identification and characterization of cellular and viral circRNAs in the context of VSV remain unexplored. In this study, we conducted a comprehensive identification and profiling of cellular and viral circRNAs following VSV infection of Vero cells, a widely used *in vitro* model for VSV. We revealed novel host-viral interactions of circRNAs: both host cellular circRNAs and viral circRNAs are induced by VSV infection, and, in turn, affected VSV infection.

## 2 Materials and methods

### 2.1 Cell and virus

Vero cells (African green monkey kidney epithelial cells), provided by the Sino-French Hoffmann Institute at Guangzhou Medical University were cultivated in Dulbecco’s minimal essential medium (DMEM; Gibco, C11965500BT) containing 10% fetal bovine serum (FBS; C04001-500) and 1% penicillin-streptomycin (final concentration: 100 U/ml penicillin and 100 ug/ml streptomycin; Gibco, 15140-122). The cells were maintained at 37°C in a humidified atmosphere containing 5% CO_2_. The vesicular stomatitis Indiana virus (VSIV) (GenBank nucleotide accession ID: NC_001560.1), hereinafter abbreviated as VSV, was also provided by the Sino-French Hoffmann Institute at Guangzhou Medical University and propagated in Vero cells for large-scale preparation. The VSV utilized in this study is the VSV-EGFP construct (12kb), in which EGFP is inserted into the G protein (GenBank accession ID: NP_041715.1). Previous studies with this VSV-EGFP strain indicate that it retains wild-type replication characteristics and does not significantly alter global transcriptional profiles (Cai et al., 2020; Yao et al., 2021; Cai et al., 2023). For viral infections, Vero cells were washed with PBS and then inoculated with the viral suspension in medium (2% FBS) for 90 minutes at 37°C at the indicated multiplicity of infection (MOI). Afterward, the inoculum was removed, and fresh medium was added.

### 2.2 VSV titration

Vero cells were cultivated until a monolayer was formed. Serial tenfold dilutions of the concentrated virus stock were prepared, ranging from 10^-1^ to 10^-7^. Three replicate wells were prepared for each dilution. Approximately 30 μL of each virus dilution was added to the corresponding wells and incubated for 2 hours at 37°C. After adsorption, an overlay medium was added to each well. The infected cells were incubated for approximately 48 hours. The overlay medium was then removed, and the cells were washed with PBS (Gibco, 10010-023). The plaques were delineated as discrete clusters of fluorescence, each consisting of at least three fluorescent foci and visualized using an inverted fluorescence microscope. The viral titer was calculated using the following formula: number of plaques/dilution factor × (1 ml/inoculated volume).

### 2.3 RNA isolation

Total RNA was extracted from cells using an RNA rapid extraction kit (GOONIE, 400-100-100T) following the manufacturer’s instructions. Nuclear RNA and cytoplasmic RNA were isolated using the PARIS Kit (Invitrogen, AM1921) according to the supplier’s protocol.

### 2.4 Library construction and RNA-sequencing

Total RNA was extracted from Vero cells under three conditions: Mock, 12 hours post-infection (hpi), and 24 hpi. To enrich for circRNAs, total RNA was subjected to ribosomal RNA (rRNA) removal, followed by treatment with RNase R to degrade linear RNAs. Subsequently, strand-specific RNA libraries were constructed and sequenced using the Illumina HiSeq 4000 platform. Library preparation and sequencing were carried out by Gene Denovo Biotechnology Co. (Guangzhou, China).High-quality clean reads were obtained by filtering reads using fastp (Chen et al., 2018) (version 0.18.0). The parameters were as follows: 1) removal of reads containing adapters; 2) removal of reads with more than 10% of unknown nucleotides; 3) removal of low-quality reads with more than 50% of low-quality (Q-value ≤ 20) bases. The rRNA-depleted reads from each sample, obtained using Bowtie2 (Langmead and Salzberg, 2012) (version 2.2.8), were then aligned to the reference genome using HISAT2 (Kim et al., 2015) (version 2.1.1). CircRNA identification and quantification were performed using CIRIquant software. A circRNA was called when it was supported by the following criteria: 1) at least one sample with ≥ 2 back-splicing reads; 2) circRNA length ≤ 100K.

### 2.5 RNase R resistance analysis

A total of 10 μg RNA was incubated for 10 minutes at 37°C, with or without the presence of 2 units of RNase R (Epicentre, RNR07250) for each microgram of RNA. Subsequently, the resulting RNA was purified using an RNeasy MinElute Cleanup Kit (Qiagen, 74204).

### 2.6 siRNA design and transfection

For the top 10 host-derived and top 10 virus-derived circRNAs identified in this study, siRNAs were designed by IGE Biotechnology Co., Ltd. (Guangzhou, China) using the DSIR online tool (http://biodev.extra.cea.fr/DSIR/DSIR.html) or by Ribo Biotechnology Co., Ltd. (Guangzhou, China), and were synthesized by these companies. The design specifically targeted the back-splice junction sequences of the circRNAs. The parameters for design included a siRNA length of 19 nt, a score threshold of 90, exclusion of sequences with ≥4 consecutive identical nucleotides, and avoidance of known immunostimulatory motifs.

For transfection, Vero cells were seeded into 6-well plates at a density of 5 × 10^5^ cells per well. When cells reached 70–80% confluence, transfection was carried out using Lipofectamine RNAiMAX (Invitrogen, 13778150), following the manufacturer’s instructions. Briefly, siRNAs were diluted to a working concentration of 10 μM and maintained on ice to prevent degradation. For each well, 9 μL of Lipofectamine RNAiMAX and 3 μL of siRNA were separately diluted in 150 μL of Opti-MEM (Gibco, 31985070), then combined at a 1:1 ratio and incubated for 5 minutes at room temperature to form siRNA–lipid complexes. After replacing the culture medium, approximately 260 μL of the transfection mixture was gently added dropwise to each well. The plates were gently swirled and incubated at 37°C in a 5% CO_2_ incubator for 72 hours, after which the cells were collected for downstream experiments.

### 2.7 Preparation of cell extracts, Western blotting and antibodies

Proteins were extracted from cells using RIPA buffer containing protease inhibitors (Sigma). Protein concentrations were determined using a BCA assay kit (Thermo Scientific). For Western blotting, equal quantities of protein extracts were boiled with a sodium dodecyl sulfate (SDS) loading buffer. The protein extracts were then subjected to SDS-polyacrylamide gel electrophoresis, and subsequently transferred to polyvinylidene difluoride (PVDF) membranes (Merck Millipore). The membrane was then incubated with the respective primary antibodies. Horseradish peroxidase (HRP)-conjugated secondary antibodies were utilized with an enhanced chemiluminescence detection system (Millipore) to visualize the proteins. The following primary antibodies used in our study: polyclonal rabbit anti-VSV G protein antibody (Abcam, ab1874) and monoclonal mouse anti-GAPDH (Proteintech, AF0006). GAPDH was used as an internal control.

### 2.8 Reverse transcription, PCR, and real-time PCR

RNA reverse transcription was carried out with random hexamers in a 20 µl reaction mixture using HiScript^®^ III All-in-one RT SuperMix Perfect for qPCR (Vazyme, R333). The reaction program was as follows: 50°C for 15 minutes, followed by 85°C for 5 seconds. The resulting cDNA was then utilized for PCR or qPCR with Taq Pro Universal SYBR qPCR Master Mix (Vazyme, Q712). Each 20 µl reaction mixture consisted of 10 µl of 2× Master Mix, 0.4 µl of each forward and reverse primer (10 µM; final concentration 0.2 µM), 2 µl of cDNA, and nuclease-free water. The cycling conditions were as follows: initial denaturation at 95°C for 30 seconds, followed by 35 cycles (PCR) or 40 cycles (qPCR) of 95°C for 10 seconds and 60 °C for 30 seconds. A melt-curve step was performed to confirm specificity, consisting of 95°C 15 seconds, 60°C 60 seconds, and 95°C 15 seconds. A list of the primers utilized in this study is provided in **Supplementary Table S1**. GAPDH, HPRT1, and ACTB were used as an internal control. Reactions were performed in triplicate using either the Applied Biosystems 7500 Real-Time PCR System or CFX Opus 96 Real-Time PCR System.

### 2.9 Differential expression analysis and time-series analysis to identify VSV-associated circRNAs

To identify differentially expressed circRNAs, the edgeR package (Robinson et al., 2010) (version 3.12.1) (http://www.r-project.org/) was used. Given that our study included two biological replicates per condition due to resource constraints, we employed edgeR’s specialized methods for small samples to improve statistical rigor. The analysis was conducted using the TMM normalization method. Dispersion estimates were calculated using a combination of common dispersion and tagwise dispersion. Exact tests between two groups were performed for statistical comparisons. The resulting P values were adjusted for multiple testing using the Benjamini-Hochberg (BH) method to control the false discovery rate (FDR). We focused on differentially expressed circRNAs with a fold change (FC) > 2 or < 0.5 and P value < 0.05. We performed Series Test of Cluster analysis using the Short Time-series Expression Miner (STEM) software on the union of differentially expressed circRNAs identified across all comparison groups. Expression values were first log2-transformed after normalization to the first time point: the expression at each time point was divided by that of the initial time point to obtain fold change, followed by log2 transformation. The clustering parameters included a minimum correlation of 0.7 and a maximum unit change of 1 in model profiles between time points. The maximum number of output trend modules was set to 10 by default. Trend modules with a significance of P < 0.05 were considered to be significantly enriched.

### 2.10 Bioinformatic tools

Viral circRNAs was demonstrated with integrative genomics viewer (IGV), with reference to the VSV genome (GenBank accession ID in: NC_001560.1). Venn diagrams were generated using an online analysis platform (http://bioinfogp.cnb.csic.es/tools/venny/index.html). The differential expression analysis of circRNAs, principal component analysis, and GO term and KEGG pathway functional annotation analysis were performed using the OmicShare tools (http://www.omicshare.com/tools), GO and pathway enrichment analysis were performed using the Omicsmart, a dynamic real-time interactive online platform (http://www.omicsmart.com).

## 3 Result

### 3.1 Identification and characterization of host cellular circRNAs in mock- and VSV-infected Vero cells

**Figure 1:**
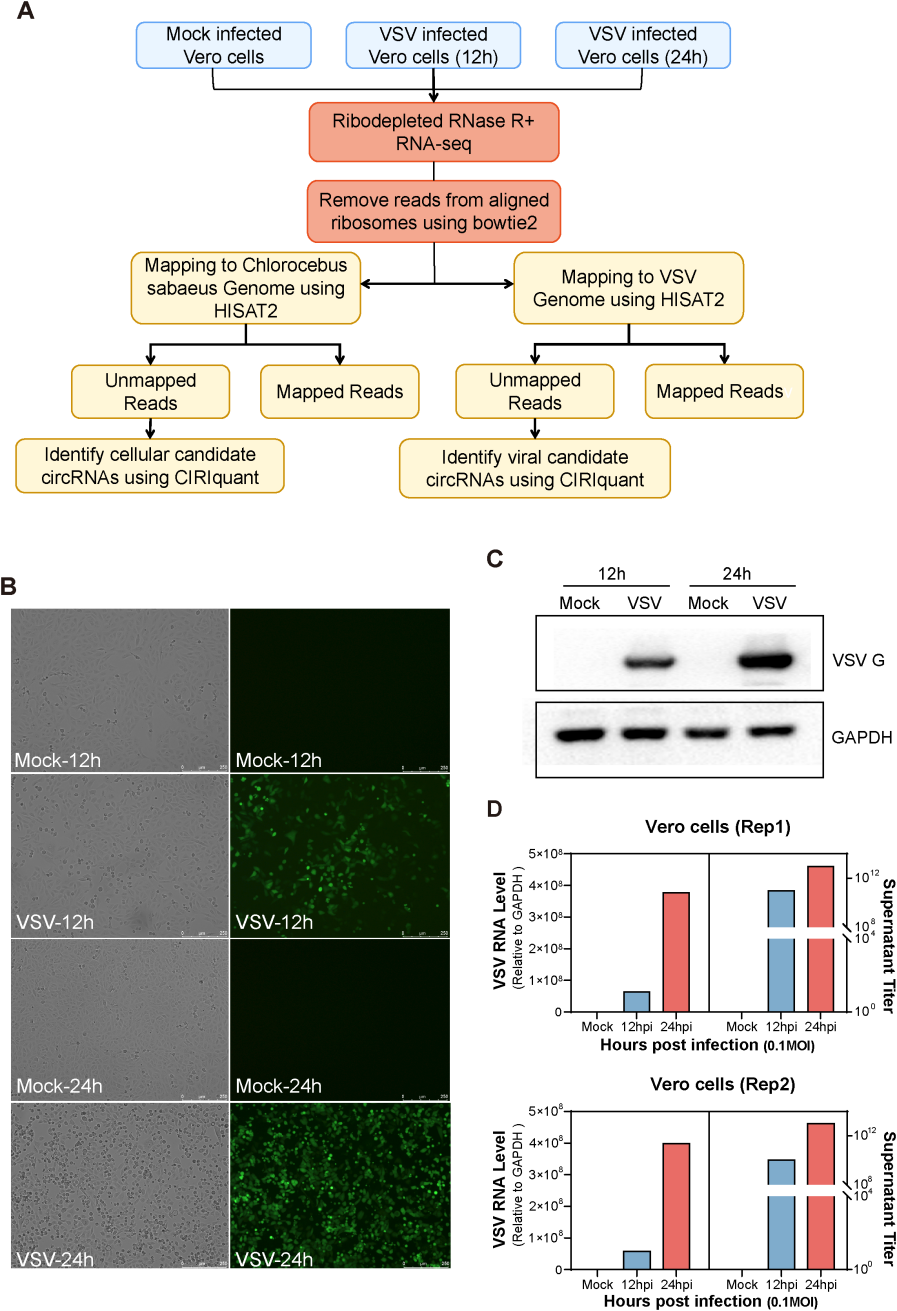
Experimental workflow and characterization of mock- and vesicular stomatitis virus (VSV)-infected Vero cells. **(A)** Schematic overview of the experimental workflow for identifying host- and virus-derived circular RNAs (circRNAs) in mock- and VSV-infected Vero cells. Total RNA was extracted from three conditions: Mock, 12 hours post-infection (hpi), and 24 hpi. Subsequently, RNA from each sample was ribodepleted and treated with RNase R. Library preparation was followed by sequencing on the Illumina HiSeq™ 4000 platform. Residual rRNAs were removed with Bowtie2, and the remaining reads were aligned to the Chlorocebus sabaeus genome and the VSV genome using HISAT2. Both host- and virus-derived circRNAs were identified using CIRIquant based on the detection of back-splice junction (BSJ) reads. **(B)** Cytopathic effects (CPE) observed in Vero cells following VSV infection. **(C)** Western blot analysis of VSV G protein expression. **(D)** Quantification of intracellular VSV RNA levels by RT-qPCR and extracellular virus titers by plaque assay. Two biological replicates were used. **(A–D)** All experiments were performed at a multiplicity of infection (MOI) of 0.1.

To characterize both host- and virus-derived cirRNA transcripts during VSV infection, we extracted total RNA from Vero cells infected with VSV at 12 and 24 hours. After removing ribosomal RNA and treating the samples with RNase R to enrich circRNAs, we conducted RNA sequencing (RNA-seq) analysis (**Figure 1A**). The MOI was 0.1, with two biological replicates per group. Mock-infected Vero cells served as a negative control (**Figure 1A**). As shown in **Figure 1B**, VSV-infected Vero cells displayed significant cytopathic effects, including cell rounding and vacuolization, along with strong GFP fluorescence expression at both 12 and 24 hours, indicating efficient viral replication. Western blot analysis further confirmed the expression of VSV G protein, which was strongly detected in infected cells at both time points, but absent in mock controls (**Figure 1C**). RT-qPCR analysis of VSV RNA levels further supported these findings, showing significantly higher viral RNA abundance in infected groups; moreover, the VSV infected Vero groups demonstrated robust virus production (**Figure 1D**). All these results confirm the successful establishment of VSV infection (**Figures 1B–D**). Each sample was sequenced using the Illumina HiSeq 4000 platform. After removing residual rRNA sequences with Bowtie2 (Langmead and Salzberg, 2012), the obtained reads were aligned to the *Chlorocebus sabaeus* (African green monkey) genome (Ensembl release 100) and the VSV genome using HISAT2 (**Figure 1A**), which employed both global and local search strategies (Kim et al., 2015). Unmapped reads were retained for subsequent circRNA identification using the CIRIquant algorithm (Zhang et al., 2020b), which allowed the detection of candidate circRNAs from both host and viral origins (**Figure 1A**).

**Figure 2:**
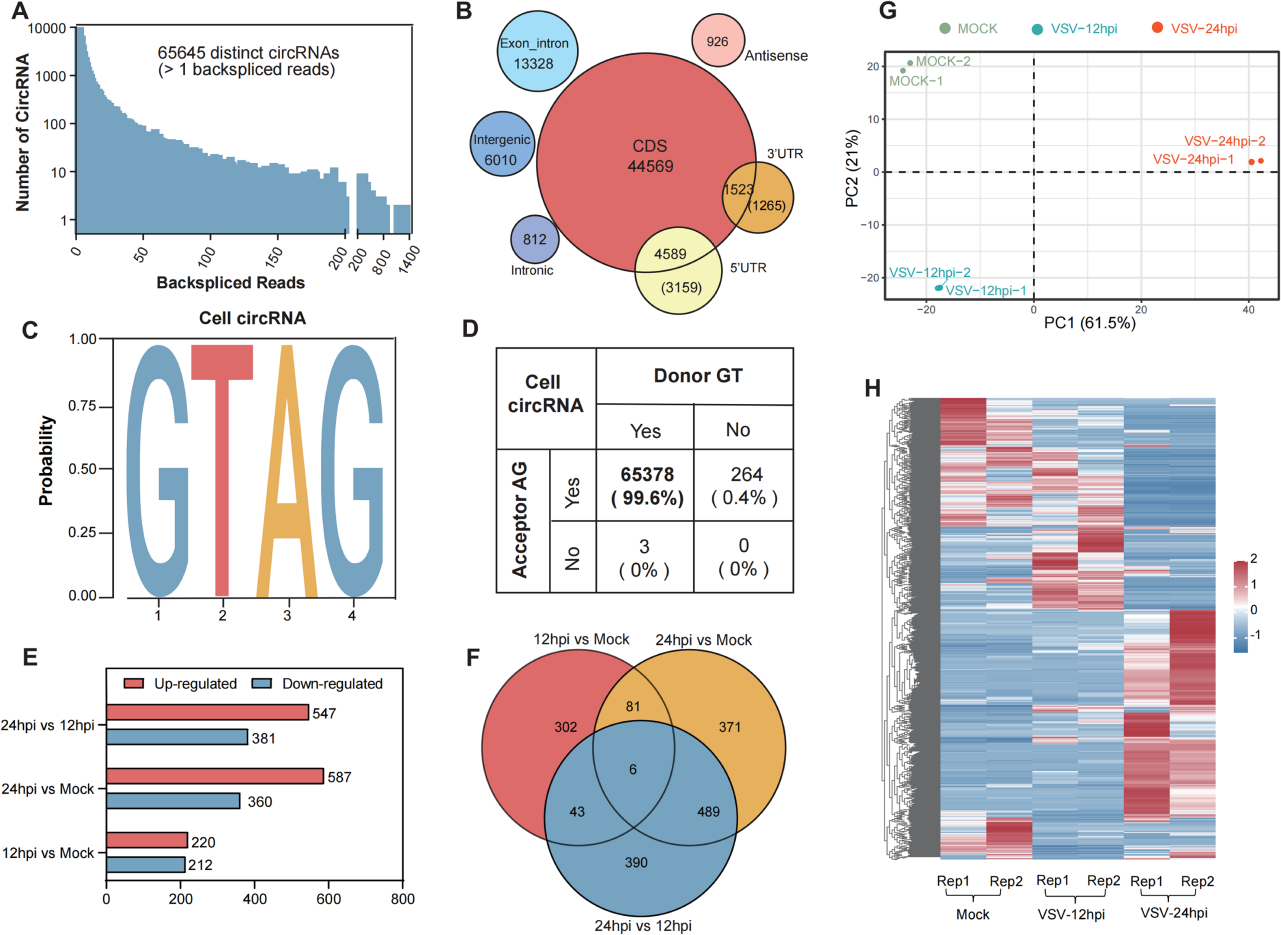
Profiling of cellular circRNAs in mock- and VSV-infected Vero cells and further differential expression analysis. **(A)** The number of cellular circRNAs identified within corresponding BSJ reads across all samples. **(B)** Genomic distribution of cellular circRNAs according to their genomic origin (including CDS, exon-intron, and intergenic), and their respective counts. **(C, D)** Summary of the flanking back-splice signals of cellular circRNAs. **(E)** Bar plot summarizing the number of differentially regulated circRNAs in each comparison groups. **(F)** Venn diagram illustrating the overlap and uniqueness of differentially expressed circRNAs among the indicated comparison groups. **(G)** Principal component analysis (PCA) based on the expression profiles of 1,682 differentially expressed cellular circRNAs. **(H)** Hierarchical clustering heatmap showing the expression patterns of 1,682 differentially expressed circRNAs across all samples. Each row represents a circRNA, each column represents a sample, and the values are log10-transformed reads per million (RPM).

All samples underwent circRNA identification and annotation, and a detailed summary of the RNA-Seq datasets for each sample is provided in **Table 1**. In total, we identified 65,645 distinct cellular circRNAs with clear back-splicing signals (**Figure 2A**). Most circRNAs exhibited low back-splice junction read support, consistent with their typically low abundance. Chromosomal mapping revealed that cellular circRNAs were distributed across all chromosomes of *Chlorocebus sabaeus*, including the sex chromosomes; chromosomes 16 and 20 harbored the highest number of circRNAs, while chromosome Y had the fewest (**Supplementary Figures S1A, B**). The average and median lengths of cellular circRNAs were 1,883 and 1,000 nucleotides (nt), respectively (**Supplementary Figure S1C**). Annotation analysis revealed that approximately 62.1% of circRNAs originated from coding DNA sequences (CDSs), while smaller proportions mapped to exon-intron, intergenic, 5’ UTRs, or 3’ UTRs (**Figure 2B**). We further analyzed the splice site characteristics of cellular circRNAs and found that the vast majority (99.6%) exhibited canonical GT/AG donor-acceptor motifs (**Figures 2C, D**), which is consistent with previously reported features of mammalian circRNAs (Guo et al., 2014).

**Table 1:** Summary of RNA-seq data from mock- and VSV-infected Vero cells.

Sample	Total reads	Mapping ratio to Chlorocebus sabaeus genome	Cellular circRNA number	Mapping ratio to VSV genome	Viral circRNA number	
Mock_*Rep1*	84,249,090	86.77%	25,823	0.00%	0	
12 hpi_*Rep1*	76,602,868	90.23%	28,802	25.00%	30	
24 hpi_*Rep1*	60,915,214	89.68%	15,825	40.83%	49	
Mock_*Rep2*	79,682,468	88.65%	25,681	0.00%	0	
12 hpi_*Rep2*	73,015,932	90.59%	25,303	29.17%	35	
24 hpi_*Rep2*	64,881,650	89.40%	15,129	29.17%	35	
Total		65,645		120		

Rep, repetition.

The table summarizes the total clean reads, mapping ratio to either *Chlorocebus sabaeus* or VSV genome, and the number of cellular or viral circRNA identified for each RNA-seq sample.

### 3.2 Differential expression analysis of cellular circRNAs in mock- and VSV-infected Vero cells

To further assess circRNA expression changes, we calculated expression levels in mock- and VSV-infected Vero cells based on RPM (back-spliced junction Reads Per Million mapped reads). Differential expression analysis was performed using the edgeR package (Robinson et al., 2010), and circRNAs with *P <* 0.05 and |log_2_ FC| > 1 were considered significantly differentially expressed. A total of 2,307 differentially regulated circRNAs were identified across the three groups (**Figure 2E**). The distribution and number of upregulated and downregulated circRNAs were presented in volcano plots (**Supplementary Figure S1D**) and bar charts (**Figure 2E**), respectively. As shown in **Figure 2E**, the 24 hpi VSV group exhibited the highest number of differentially expressed circRNAs compared to the mock group, with 587 circRNAs upregulated and 360 downregulated; when comparing the 24 hpi group to the 12 hpi group, 547 upregulated and 381 downregulated circRNAs were identified; in contrast, the comparison between the 12 hpi group and the mock group revealed 220 upregulated and 212 downregulated circRNAs.

Venn diagram analysis showed partial overlap in circRNA expression profiles between different groups. Specifically, 6 circRNAs were commonly differentially expressed across all three groups, while pairwise comparisons identified 81, 43, and 489 shared circRNAs, respectively (**Figure 2F**). By combining data from **Figures 2E, F**, a total of 1,682 unique differentially expressed circRNAs were identified. Principal component analysis (PCA) of these circRNAs, based on the three main sources of variance (PC1, PC2, and PC3), revealed clear separation between the mock, 12 hpi, and 24 hpi groups, indicating that VSV infection substantially alters host circRNA expression profiles (**Figure 2G**). Hierarchical clustering heatmap analysis of these circRNAs revealed three major sample clusters corresponding to the Mock, 12 hpi, and 24 hpi groups (**Figure 2H**) and the distinct expression patterns within these clusters suggest that specific circRNA groups may be co-regulated during infection. These findings indicate strong reproducibility and highlight a profound and consistent induction of the host circRNAome in response to VSV infection over time.

### 3.3 Trend analysis with differentially expressed circRNAs and further functional annotation

**Figure 3:**
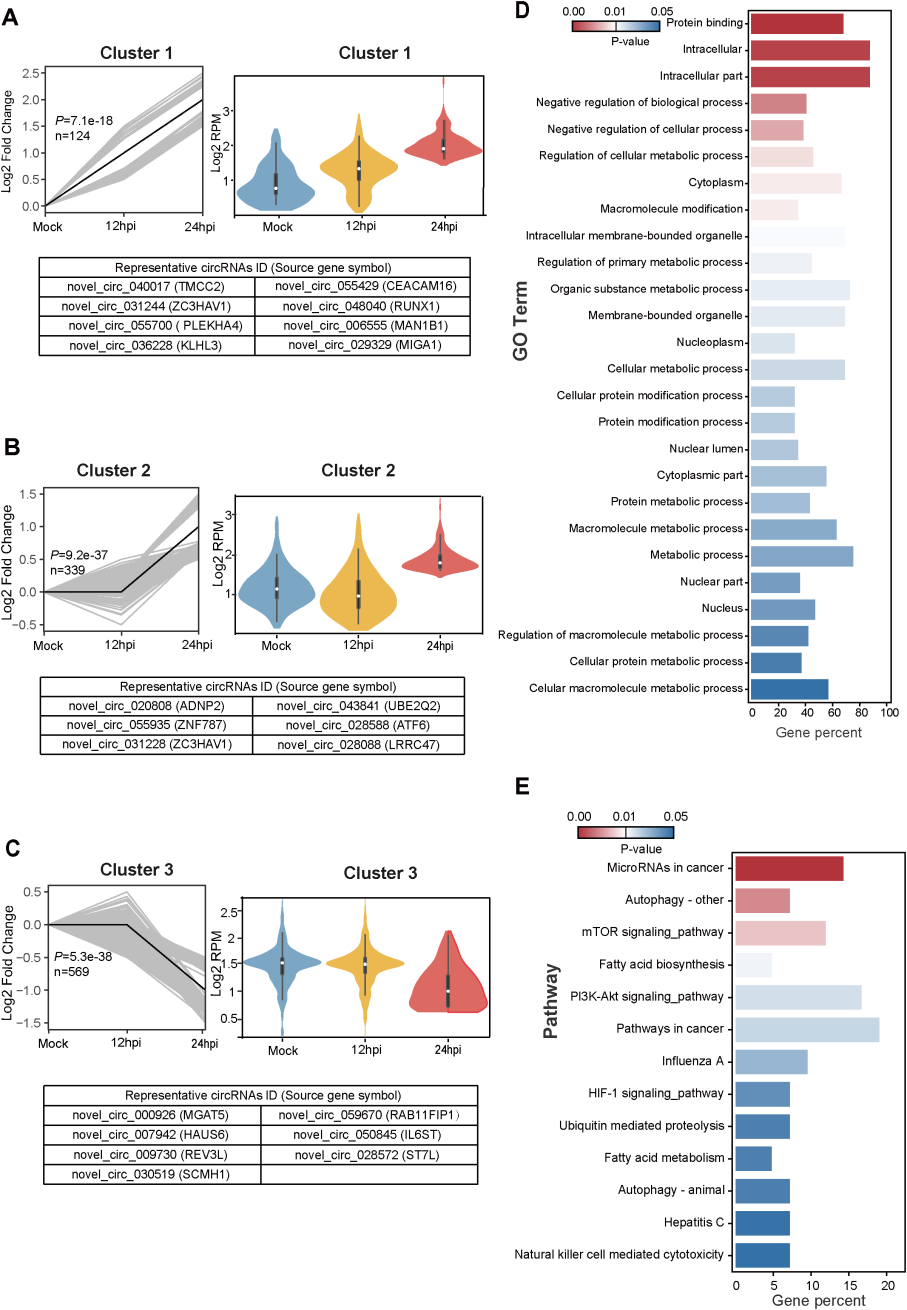
Temporal expression patterns of differentially expressed circRNAs and subsequent functional enrichment analysis. **(A–C)** Trend analysis of 1,682 differentially expressed circRNAs performed using Short Time-series Expression Miner (STEM). Three expression clusters were identified as statistically significant (*P <* 0.05). Left panels: Line plots show expression trends of circRNAs within each cluster. Gray lines represent individual circRNA expression profiles while the black line indicates the model trend for each cluster. The number of circRNAs assigned to each cluster and their corresponding P-values are provided. Right panels: Violin plots depict the absolute expression levels (log_2_ scale) of circRNAs within each cluster. Bottom panels: Representative circRNAs for each cluster are listed, with the corresponding source gene symbols in parentheses. **(D, E)** Functional enrichment analysis of the protein-coding parental genes of circRNAs from cluster 1. **(D)** Gene Ontology (GO) enrichment analysis results. **(E)** Kyoto Encyclopedia of Genes and Genomes (KEGG) pathway enrichment analysis results.

To further elucidate the dynamic expression pattern of cellular circRNAs during VSV infection, we performed short time-series expression miner (STEM) clustering analysis (Ernst and Bar-Joseph, 2006). Among the 1,682 differentially expressed circRNAs identified, three significant temporal expression clusters were observed (*P <* 0.05; **Figures 3A–C**), which exhibit continuous induction trend across infection time points, as well as five clusters that did not reach statistical significance (**Supplementary Figure S2**). Line graphs and violin plots depicted the fold change trends and normalized RPM expression levels, respectively (**Figures 3A–C**; **Supplementary Figure S2**). The three significant clusters contained 124, 339, and 569 circRNAs, respectively, with representative circRNAs of each cluster shown in the lower panels (**Figures 3A–C**).

We next performed Gene Ontology (GO) and Kyoto Encyclopedia of Genes and Genomes (KEGG) pathway enrichment analyses with parental protein-coding genes of circRNAs (Huang da et al., 2009). In the analysis, we focused on the continuously increasing cluster 1, which comprised a total of 124 cellular circRNAs from 102 parental source genes. We screened 26 regulated GO terms (*P <* 0.05) (**Figure 3D**) and 13 regulated KEGG pathways (*P <* 0.05) (**Figure 3E**), which were displayed and ranked by their P values. The 102 genes showed significant enrichment of GO processes related to Macromolecule modification, Regulation of cellular metabolic process, and Membrane/Intracellular membrane-bounded organelle (**Figure 3D**). These genes also showed significant enrichment of KEGG pathways associated with mTOR signaling pathway, Fatty acid biosynthesis/metabolism, PI3K-Akt signaling pathway, Influenza A, and Hepatitis C (**Figure 3E**). In conclusion, GO and KEGG pathway enrichment analysis suggest that circRNAs may be associated with pathways related to virus infection, molecule modification, cellular metabolism, and signaling regulation.

### 3.4 Validation of the top 10 cluster 1-derived cellular circRNAs and their VSV-induced upregulation

**Figure 4:**
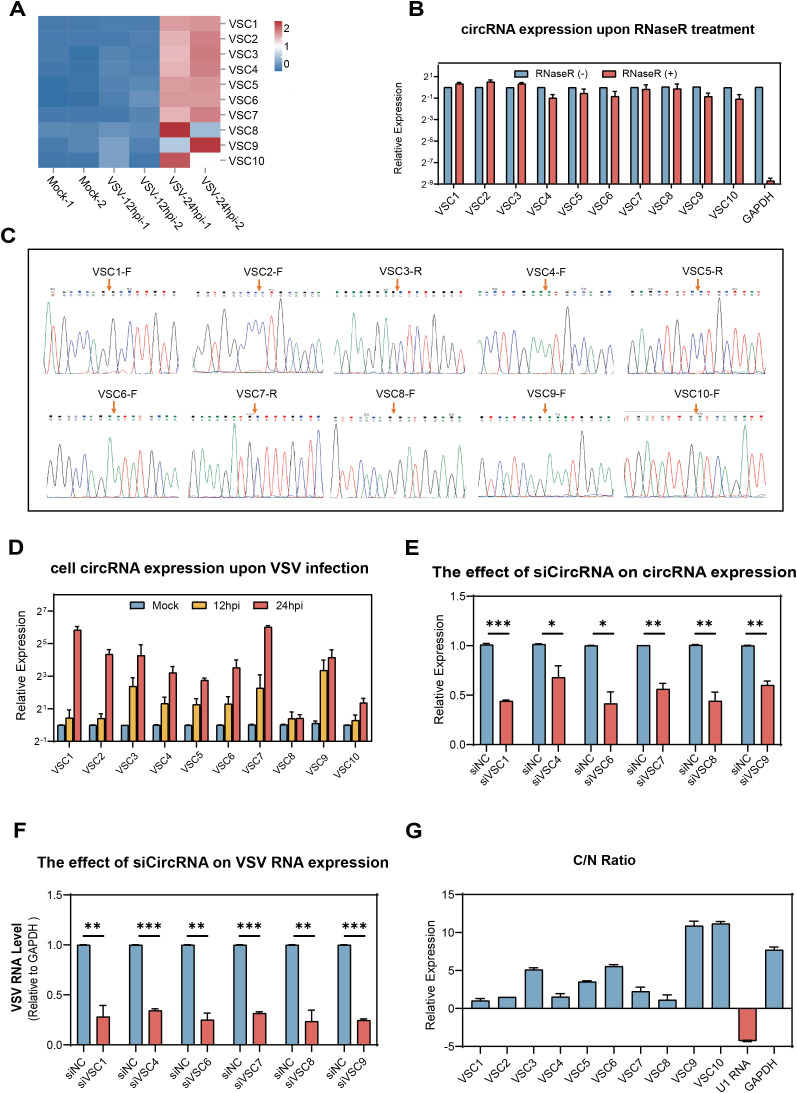
Validation of the top 10 VSV-stimulated circRNAs (VSCs) and analysis of their effects on viral replication and their subcellular localizationVero. **(A)** A heatmap showing the expression profiles of the top 10 VSCs, based on normalized RPM values from RNA-seq data (log_10_ scale). **(B)** RT-qPCR validation of RNase R resistance of the top 10 VSCs. Expression levels were normalized to the untreated control group. **(C)** Sanger sequencing of the PCR product using divergent primers to detect BSJs of the VSCs. Red arrows indicate the position of the BSJs. **(D)** RT-qPCR analysis of the expression levels of the top 10 VSCs in mock- and VSV-infected cells (0.1 MOI), with expression normalized to the housekeeping gene GAPDH and are presented relative to the mock-infected group. **(E)** RT-qPCR analysis of circRNA expression in Vero cells transfected with siRNAs targeting individual circRNAs. **(F)** RT-qPCR quantification of intracellular VSV RNA levels following knockdown of each VSC (excluding VSC2, VSC3, VSC5, and VSC10) by siRNA transfection, with data normalized to the negative control (siNC). *P < 0.05; **P < 0.01; ***P < 0.001; NS, no significance. **(G)** Subcellular localization analysis of the 10 VSCs in nuclear and cytoplasmic fractions of Vero cells using RT-qPCR, with expression levels normalized to the nuclear fraction. C, cytoplasm; N, nuclear. **(B, D–G)** Data are presented as mean ± SEM (n = 3).

For further analysis, we focused on the circRNAs from cluster 1 (*P* = 7.1e-18), which exhibited a steady and significant upregulation during VSV infection. The top 10 ranked circRNAs were screened out and referred to as VSV-stimulated circRNAs (VSCs), were selected based on high expression values at both 12 and 24 hpi and the magnitude of their upregulation, using the following FC cutoffs to ensure robust and dynamic responders: a > 30-FC when comparing the 24 hpi group to the mock group and a > 7-FC when comparing the 24 hpi group to the 12 hpi group. A detailed characterization of the 10 VSCs is listed in **Table 2**. Heatmap analysis demonstrated that VSV infection resulted in significant changes in VSC expression, especially at 24 hpi, with several VSCs exhibiting time-dependent expression patterns (**Figure 4A**). To confirm the presence of VSCs, we designed divergent primers (**Supplementary Table S1**) for the 10 VSCs and performed PCR with VSV-infected Vero cells. The results showed that each primer set successfully amplified a single product of the expected size (**Supplementary Figure S3A**). The accuracy of the back-splicing site was confirmed by Sanger sequencing (**Figure 4C**). Additionally, we assessed circRNA stability following RNase R treatment, and the results showed that the 10 VSCs remained highly stable, whereas the control linear GAPDH mRNA was significantly degraded (**Figure 4B**). These findings collectively verify the authenticity of the circRNAs.

**Table 2:** Cellular and viral circRNAs validated in this study.

Alias name	CircRNA ID	Position	Strand	Genomic length	Spliced length	Gene symbol
VSC1	novel_circ_040017	25:24063696-24082000	+	18,305	18,305	TMCC2
VSC2	novel_circ_055429	6:38187008-38187334	+	327	327	CEACAM16
VSC3	novel_circ_031244	21:107803964-107808367	-	4,404	3,951	ZC3HAV1
VSC4	novel_circ_048040	2:78890978-78918679	-	27,702	516	RUNX1
VSC5	novel_circ_055700	6:42092405-42106835	-	14,431	8,024	PLEKHA4
VSC6	novel_circ_031229	21:107785437-107804216	-	18,780	2,438	ZC3HAV1
VSC7	novel_circ_015212	15:86835919-86856982	-	21,064	21,064	/
VSC8	novel_circ_006555	12:1094123-1102999	-	8,877	398	MAN1B1
VSC9	novel_circ_036228	23:40392381-40402269	-	9,889	499	KLHL3
VSC10	novel_circ_029329	20:55357410-55374690	-	17,281	700	MIGA1
vsv_circ_001	–	VSV: 10371-11885	-	1515	1515	VSV L gene
vsv_circ_007	–	VSV: 10837-11103	+	267	267	VSV N gene
vsv_circ_027	–	VSV: 212-1031	+	820	820	VSV L gene
vsv_circ_028	–	VSV: 2397-3919	-	1523	1523	VSV G gene
vsv_circ_029	–	VSV: 2429-2882	+	454	454	VSV L gene
vsv_circ_070	–	VSV: 5635-6113	+	479	479	VSV L gene
vsv_circ_077	–	VSV: 56-470	+	415	415	VSV L gene
vsv_circ_088	–	VSV: 6114-6449	+	336	336	VSV L gene
vsv_circ_105	–	VSV: 857-1313	+	457	457	VSV L gene
vsv_circ_108	–	VSV: 9028-9954	-	927	927	VSV L gene

The table provides the position of each VSV-stimulated cellular circRNA (VSC) or the top 10 viral circRNA validated in this study on the *Chlorocebus sabaeus* or VSV genome, the genomic and back-spliced length of each circRNA, and the parental source genes (gene_symbol) from which circRNAs are derived.

To confirm the effect of VSV infection on the expression levels of VSCs at different time points, we examined the dynamic changes of VSCs using RT-qPCR (MOI = 0.1). The RT-qPCR results were highly consistent with the RNA-seq data, which showed that all the 10 VSCs were significantly upregulated in VSV infected Vero cells and three of the VSCs (VSC1, VSC7 and VSC9) exhibited the strongest induction (**Figures 4D** and **S4A, B**). These finding suggest that the circRNAs may play key regulatory roles during VSV infection.

### 3.5 Effects of the top 10 VSCs on VSV replication and their subcellular localization

We further investigated whether the elevated top 10 VSCs affects VSV infection in turn. VSC2, VSC3, VSC5, and VSC8 were excluded due to sequence design limitations, such as high GC content or unfavorable junction structures. All siRNAs were designed to avoid interfering with their linear host transcripts. Vero cells were transfected with circRNA-targeting siRNAs 24 hours prior to VSV infection. Total RNA was extracted at 24 hpi for analysis. Further RT-qPCR results showed that the specific siRNAs of all the 6 VSCs efficiently knock downed their respective circRNAs (**Figure 4E**). Under these conditions, silencing all 6 circRNAs resulted in significant reductions in VSV RNA levels (**Figure 4F**), supporting a positive regulatory effect on viral replication. Subcellular fractionation followed by RT-qPCR revealed that all 6 circRNAs were predominantly localized in the cytoplasm (**Figure 4G**), which indicate a potential post-transcriptional regulatory function. U1 snRNA and GAPDH mRNA served as nuclear and cytoplasmic controls, respectively (**Figure 4G**). Together, these findings demonstrate that the 6 circRNAs from cluster 1 are enriched in the cytoplasm and promote VSV replication.

### 3.6 Identification and characterization of viral circRNAs in VSV-infected Vero cells

**Figure 5:**
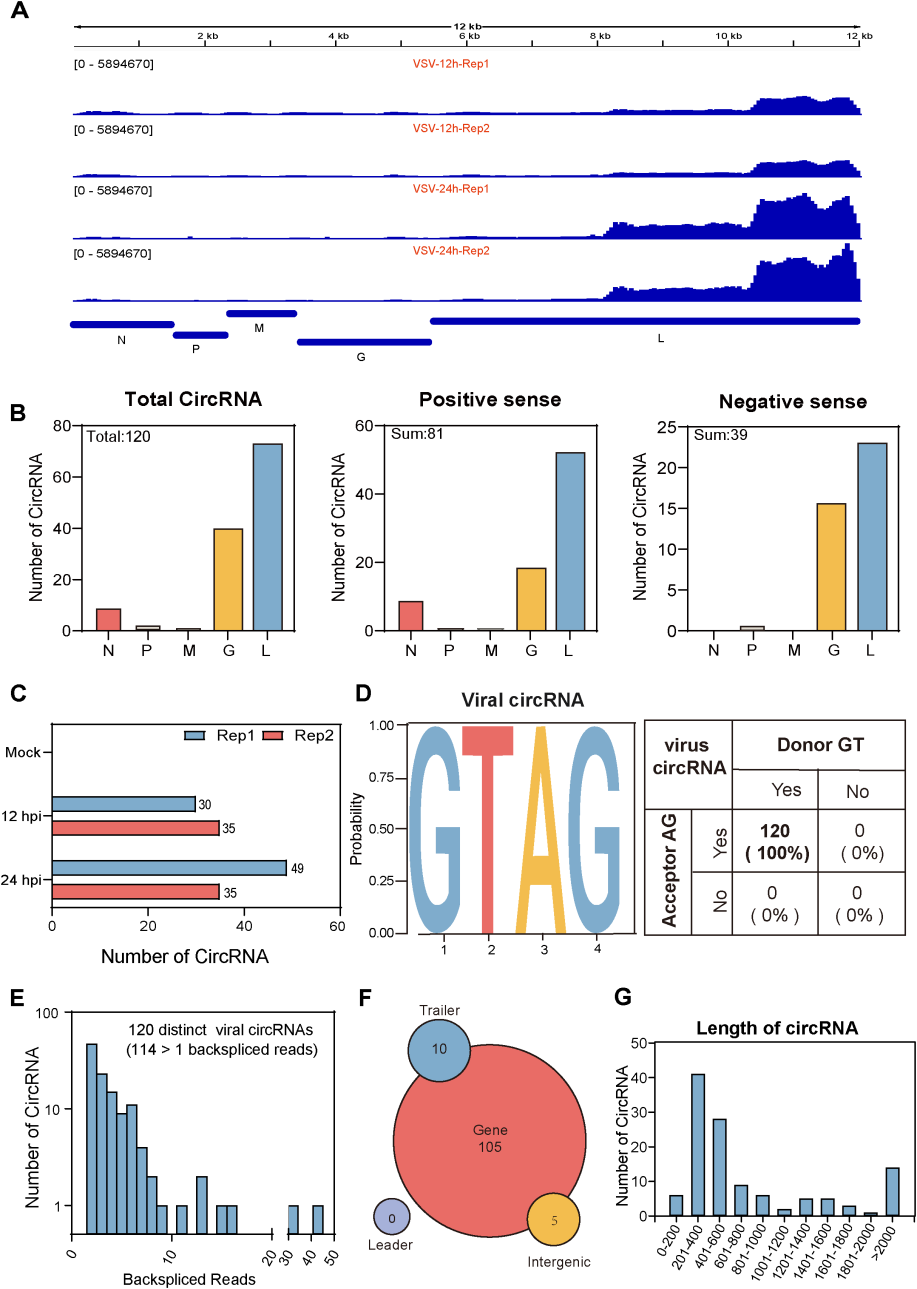
Characterization of viral circRNAs in VSV-infected Vero cells. **(A)** Visualization of viral circRNAs identified in VSV-infected samples using Integrative Genomics Viewer (IGV). The viewer displays coverage the of BSJ reads mapped to the VSV genome for each infected sample, with standardized coverage depth on the Y-axis. **(B)** The number of viral circRNAs mapped to each VSV gene, categorized by total viral RNA (left panel), positive-sense RNA (middle panel), and negative-sense RNA (right panel). **(C)** Bar plots showing the total and individual counts of viral circRNAs across all infected samples. **(D)** Summary of the flanking signals of viral circRNAs. **(E)** The number of viral circRNAs detected in the corresponding BSJ reads across all samples. **(F)** Genomic annotation of viral circRNAs based on their origin (gene mRNA, trailer, and intergenic), along with their respective counts. **(G)** Distribution of viral circRNA lengths.

In addition to studying host cell-derived circRNAs, we also investigated circRNAs derived from VSV. The pipeline for identifying cellular and viral circRNAs has been previously described in **Figure 1A**. We identified a total of 120 viral back-splicing junctions in VSV-infected samples and visualized them using the integrative genomics viewer (IGV) based on the VSV genome (Robinson et al., 2011). The coverage depth for each alignment panel is shown on the Y-axis (**Figure 5A**). **Figure 5A** respectively show that the L gene region of the VSV genome exhibited significant coverage peaks. We also analyzed the circRNA number of each VSV gene from the general VSV RNA (120 in total) and their distribution on the positive strands (81 in sum) and negative strands (39 in sum); data revealed that most of the circRNAs were concentrated in the L and G gene regions (**Figure 5B**). Additionally, the number of viral circRNAs identified from each sample is presented: 79 in the 12 hpi group and 70 in the 24 hpi group (**Figure 5C**).

We further systematically characterize viral circRNAs (**Figures 5D–G**). First, analysis of the splicing signals of viral circRNAs showed that all (100%) contained GT/AG donor-acceptor sequences (**Figure 5D**), consistent with the classical eukaryotic splicing pattern. Second, we analyzed the read distributions of viral circRNAs. Among the 120 viral circRNAs, 114 contained at least two unique back-splicing read segments (**Figure 5E**). Third, further analysis of their genomic origin revealed that nearly all viral circRNAs originated from VSV gene RNA, with only a few mapping to trailer and intergenic sequences (**Figure 5F**). No viral circRNAs aligned with the leader sequences (**Figure 5F**). Finally, we examined the length distribution of the circRNAs and found that their mean and median lengths were 860 nt and 600 nt, respectively, with most circRNAs concentrated within the 200–600 nt range (**Figure 5G**).

### 3.7 Profiling of the top 10 viral circRNAs and their effects on VSV replication

**Figure 6:**
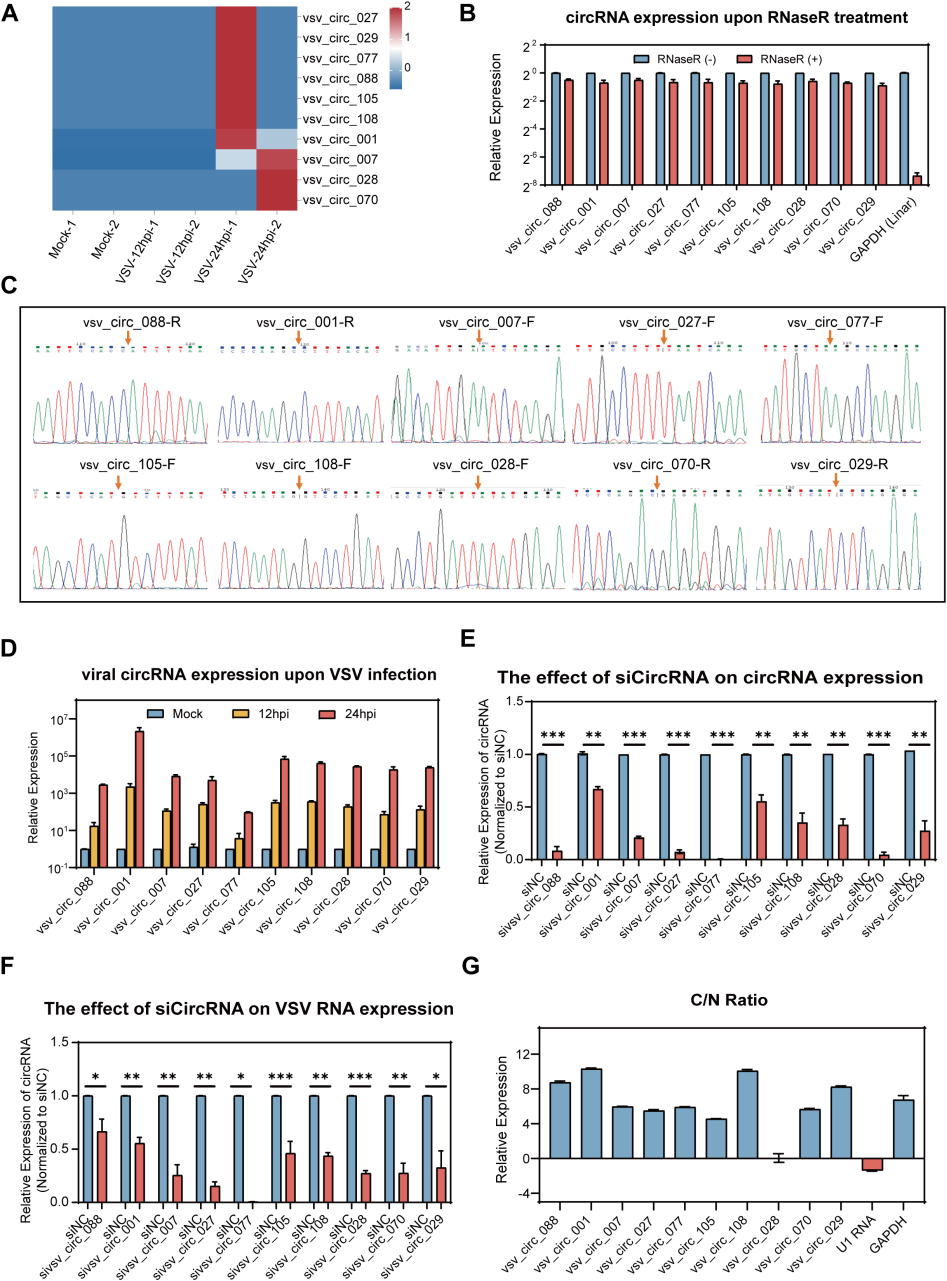
Expression profiling of the top 10 viral circRNAs and their impact on VSV replicationVero. **(A)** A heatmap illustrating the expression profiles of the top 10 VSV circRNAs (log_10_-transformed RPM). **(B)** RT-qPCR analysis of the 10 viral circRNAs following RNase R treatment. Expression levels were normalized to the untreated controls. **(C)** Sanger sequencing of the PCR product using divergent primers to detect BSJs of the 10 viral circRNAs, with red arrows marking the BSJ sites. **(D)** RT-qPCR quantification of the 10 viral circRNAs following VSV infection (0.1 MOI), with normalization to mock-infected controls. **(E)** RT-qPCR analysis of the expression of the 10 viral circRNAs in Vero cells transfected with siRNAs targeting each circRNA. **(F)** RT-qPCR quantification of VSV RNA levels upon circRNA knockdown. Data are normalized to siNC group. **(G)** Subcellular distribution of viral circRNAs in nuclear and cytoplasmic compartments, with the C/N ratio indicating the relative enrichment of cytoplasm over nucleus. **(B, D–F)** Data are presented as mean ± SEM (n = 3). *P<0.05; **P<0.01; ***P<0.001.

To further analyze the expression levels of viral circRNAs during VSV infection, we calculated RPM which was also used to cellular circRNAs for quantitative analysis. Based on their expression level, we selected the top 10 differentially expressed viral circRNAs for further investigation. The heat map of the expression of the 10 viral circRNAs were shown in **Figure 6A**, and the detailed characteristics of these circRNAs was listed in **Table 2**. We designed specific primers for these 10 viral circRNAs and performed PCR amplification in VSV-infected Vero cells. The experimental results showed that each primer set successfully amplified a single product of the expected size (**Supplementary Figure S3B**). The correct back splice sites were subsequently validated by Sanger sequencing (**Figure 6C**). Additionally, RNase R digestion experiments revealed that these 10 viral circRNAs, like cellular circRNAs, remained highly stable after digestion treatment (**Figure 6B**), further confirming their circular structures. RT-qPCR was then used to quantify the expression trend of these viral circRNAs in Vero cells after VSV infection (MOI = 0.1). The results showed that these 10 viral circRNAs were significantly upregulated upon VSV infection, with upregulations at 24 hpi exceeding more than 1,000-fold compared to the mock group (**Figure 6D**).

To explore the potential role of viral circRNAs during VSV infection, we utilized RNA interference technology for functional validation. Vero cells were transfected with siRNA mixtures prior to VSV infection, and samples were collected at 24 hpi for analysis. RT-qPCR results revealed that the specific siRNAs effectively decreased the expression levels of the corresponding circRNAs (**Figure 6E**). Silencing all the 10 viral circRNAs resulted in a significant decrease in VSV RNA levels (**Figure 6F**), suggesting that these circRNAs positively contribute to VSV infection. Additionally, we analyzed the subcellular localization of these 10 viral circRNAs. As shown, 9 of the circRNAs were mainly enriched in the cytoplasm, while vsv_circ_028 was distributed in both the cytoplasm and the nucleus (**Figure 6G**). These findings suggest that all the 10 viral circRNAs, primarily localized in the cytoplasm, exert proviral functions.

## 4 Discussion

This study is the first to systematically investigate the cellular and viral cyclic RNAs induced by VSV infection of host Vero cells, which is a widely used *in vitro* model for VSV and the most commonly accepted continuous cell line by regulatory authorities used for vaccine production (Barrett et al., 2009). On the one hand, from the perspective of host cell circRNAome, we identified a large number of circRNAs in both the mock and VSV infected Vero cells, originating from different genome locations. Screening analysis identified 1,679 differentially expressed circRNAs, and further STEM trend clustering with these circRNAs identified three significant expression clusters (cluster1, cluster2, and cluster3). Subsequently, 124 circRNAs from the steadily increasing cluster 1 were analyzed for GO process and KEGG pathway enrichment of the parental source genes. Next, the existence of the top 10 VSCs in the focused cluster1 was verified by RT-PCR, Sanger sequencing, and RNase R resistance. And the upregulated expression of these 10 VSCs following VSV infection of Vero cells was verified using RT-qPCR. In addition, siRNA results showed that these VSCs generally enhanced VSV replication. On the other hand, from the perspective of the viral circRNAome, we identified 120 viral circRNAs in VSV-infected Vero cells. The top 10 viral circRNAs were screened, and the authenticity of these viral circRNAs was verified by the same assays for cellular circRNAs: single RT-PCR product, Sanger sequencing, and RNase R resistance experiments. The induced expression of the 10 circRNAs by VSV infection was validated by RT-qPCR in Vero cells. In addition, siRNA results indicated that these viral circRNAs promoted VSV replication as well.

CircRNAs are found in all domains of life and have been identified and characterized in both eukaryotic cells and viruses. The present study comprehensively profiled the host- and virus-derived circRNAs during VSV infection in Vero cells, further extending the range of eukaryotic cells and viruses. We systematically analyzed the circRNA profiles, identifying 65,645 cellular circRNAs from the *Chlorocebus sabaeus* genome (3.2 Gb) and 120 viral circRNAs from the VSV RNA genome (12 Kb). The smaller VSV RNA genome appears to produce more circRNAs per kilobase of genome length compared to the *Chlorocebus sabaeus* genome, indicating a higher density of circRNA production in the viral genome. Further characterizations of both cellular and viral circRNAs were performed. First, genomic locations were analyzed, and result showed that most host circRNAs were derived from exon cyclization whereas VSV viral circRNAs were almost exclusively derived from the L gene region, suggesting the specificity of the generation mechanism. Second, length analysis showed that the mean (1,883 nt) and median (1,000 nt) lengths of cellular circRNAs were higher than those (860 nt and 600 nt respectively) of viral circRNAs, suggesting that virus derived circRNAs have a more concise structural character. Third, analysis of back-splicing signals showed that the splice donor/acceptor sites of both host and virus-derived circRNAs almost always conformed to the GT/AG rule, suggesting a similarity in the synthesis of cellular and viral circular RNAs, possibly relying on the host cellular spliceosome mechanism (Li et al., 2020).

In the current study, we found that VSV infection induced significant changes in host cellular circRNA expression. And as demonstrated, the number of upregulated circRNAs exceeded that of downregulated circRNAs (**Figure 2E**). By characterizing the dynamic changes of cellular circRNAs upon VSV infection, we identified 124 circRNAs with VSV-induced steadily increasing expression patterns, designated as cluster 1. And VSV infection upregulated expression of the top 10 VSCs from cluster 1. Notably, some parental genes of these VSCs have been reported to be elevated by viral infection. For instance, *ZC3HAV1* (the parental gene of VSC3 and VSC6) was significantly upregulated by infection with influenza A virus (IAV) and Sendai virus (Zhang et al., 2020a). Similarly, *RUNX1* (the parental gene of VSC4) was elevated by IAV infection (Hu et al., 2022). These findings suggest that these VSCs share the same expression regulation pattern as their parental genes. Further functional annotation revealed that their parental genes were significantly associated with various biological themes and signaling pathways, suggesting that they may be synergistically involved in viral replication through these biological processes. Interestingly, many viruses are reported to activate the identified GO and KEGG pathway to promote viral replication, such as PI3K-Akt signaling_pathway, Fatty acid biosynthesis, Macromolecule modification and Cellular protein modification process (Dunn et al., 2009; Abe et al., 2020; Zhu and Zheng, 2020; Jani et al., 2025). Functional experiment using siRNAs targeting the top 10 VSCs demonstrated that these cellular circRNAs generally enhanced VSV replication, which is reasonable and supports the assertion that these circRNAs are associated with the proviral response induced by VSV infection, as indicated by the GO and KEGG pathway enrichment analysis. Subsequent investigation demonstrated that the top 10 VSCs were predominantly localized in the cytoplasm. Cytoplasmic circRNAs are reported to function through mechanisms such as microRNA sponging and even protein-encoding capability. Whether the VSCs function through these mechanisms is an intriguing question that warrants further investigation.

Besides cellular circRNAs, we identified a group of viral circRNAs with distinct origins during VSV infection. A total of 120 VSV derived circRNAs were identified, and this identification, along with previous studies (Wongsurawat et al., 2019; Kakuk et al., 2021), adds a new layer to the growing understanding of VSV transcriptomic complexity. The fact that LRS can capture > 95% of VSV genome in single reads (Wongsurawat et al., 2019) further supports the feasibility of detecting full-length circRNAs from the viral genome. Among the 120 VSV circRNAs, 81 was derived from positive-sense antigenomic or mRNAs, more than the 39 negative-sense stranded circRNAs. This phenomenon is consistent with previous studies that the abundance of antigenomic RNAs and mRNAs typically exceeds that of genomic RNAs during VSV infection (Whitlow et al., 2006; Neidermyer and Whelan, 2019). Further analysis revealed that these viral circRNAs primarily originated from L and G genes in the VSV genome, which consists of five genes arranged in the order of N-P-M-G-L. Among these five genes, L is the largest gene and most conserved throughout Rhabdovirida, playing a central role in viral replication and transcription. Our data demonstrated that L was the most abundant region for circRNA production, suggesting a sequence or structural advantage in circRNA biogenesis (Morin et al., 2012); this finding intriguingly contrasts with the low expression levels of L gene transcripts reported by Kakuk et al. in glioblastoma and fibroblast cells (Kakuk et al., 2021), highlighting a potential divergence between circular and linear RNA production across cell types. It is noted that our study with short-read circRNA-seq was conducted in Vero cells, whereas the long-read transcriptomic studies by Kakuk et al. utilized glioblastoma and fibroblast cells. Future studies using multi-omics approaches in unified experimental systems will be valuable to investigate cell-type-specific profiles of both linear and circular viral transcripts. In addition, it is noteworthy that the expression levels of certain VSV derived circRNAs were comparable to, or even exceeded, those of highly expressed cellular circRNAs, suggesting their stable production during VSV infection and potential functional significance (Chen, 2020). Moreover, siRNA results revealed that silencing the top 10 viral circRNAs significantly decreased VSV RNA levels, further supporting their involvement in the viral life cycle or host response regulation. In conclusion, our findings identify a set of VSV circRNAs with potential functional roles in VSV infection.

Our study with siRNA knockdown experiments demonstrates that both host and viral circRNAs promote VSV replication, although their mechanisms of action remain to be elucidated. Future studies could investigate whether these circRNAs influence VSV infection or host immune responses through canonical mechanisms such as acting as sponges to sequester miRNAs, interacting with RNA-binding proteins to modulate mRNA stability or transcription, or even encoding small peptides that affect infection. Furthermore, it would be valuable to examine whether these circRNAs are packaged into exosomes and secreted, potentially mediating paracrine communication to neighboring cells, thereby influencing viral infection. This study primarily focused on functionally validating the top 10 host and top 10 viral circRNAs. However, analysis of the remaining candidates shows that many exhibit similar temporal expression patterns, share predominant cytoplasmic localization, and conserve key genomic features, including canonical GT/AG splicing signals. Furthermore, the high enrichment of viral circRNAs originating from the L gene suggests a potential common biogenesis mechanism or functional preference. While further experimental validation is required, the consistent genomic features and expression dynamics across the broader circRNA population support the conclusion that circRNA-mediated regulation plays a significant and widespread role in the host-VSV interaction. Although this study focuses on VSV, the induction of proviral circRNAs is likely a broader strategy employed by other RNA viruses as well. Our research, along with emerging identification of functional circRNAs that regulate viral replication or modulate host responses across diverse RNA viruses (Yao et al., 2021; Zhang et al., 2022; Cao et al., 2024), suggests that circRNA-mediated regulation may represent a common layer of virus-host interaction. These findings open promising therapeutic avenues: the sensitivity of VSV replication to circRNA knockdown positions both host and viral circRNAs as potential targets for novel RNA-based antivirals, while their stability and specific expression patterns support their development as diagnostic or prognostic biomarkers for viral infection.

In summary, we identified a novel aspect of host-viral interactions involving circRNAs: VSV infection led to the differential expression of both cellular and viral circRNAs, which subsequently influenced VSV infection. These cellular and viral circRNAs could serve as novel biomarkers or therapeutic targets, providing a foundation for developing innovative anti-VSV strategies. Additionally, VSV is extensively used in both basic research and medical applications, including vaccine development, while Vero cells are commonly employed in vaccine production. This study’s comprehensive circRNA profiling during VSV infection in Vero cells may provide valuable insights for these fields from a circRNA perspective.

## Data Availability

The raw data supporting the conclusions of this article will be made available by the authors, without undue reservation.
