# Supplementary figures and images for: Comprehensive profiling of host- and virus-derived circular RNAs during vesicular stomatitis virus infection
Source: Front Cell Infect Microbiol. 2025 Oct 16;15:1654185. doi: 10.3389/fcimb.2025.1654185 (PMC12571801; doi:10.3389/fcimb.2025.1654185)

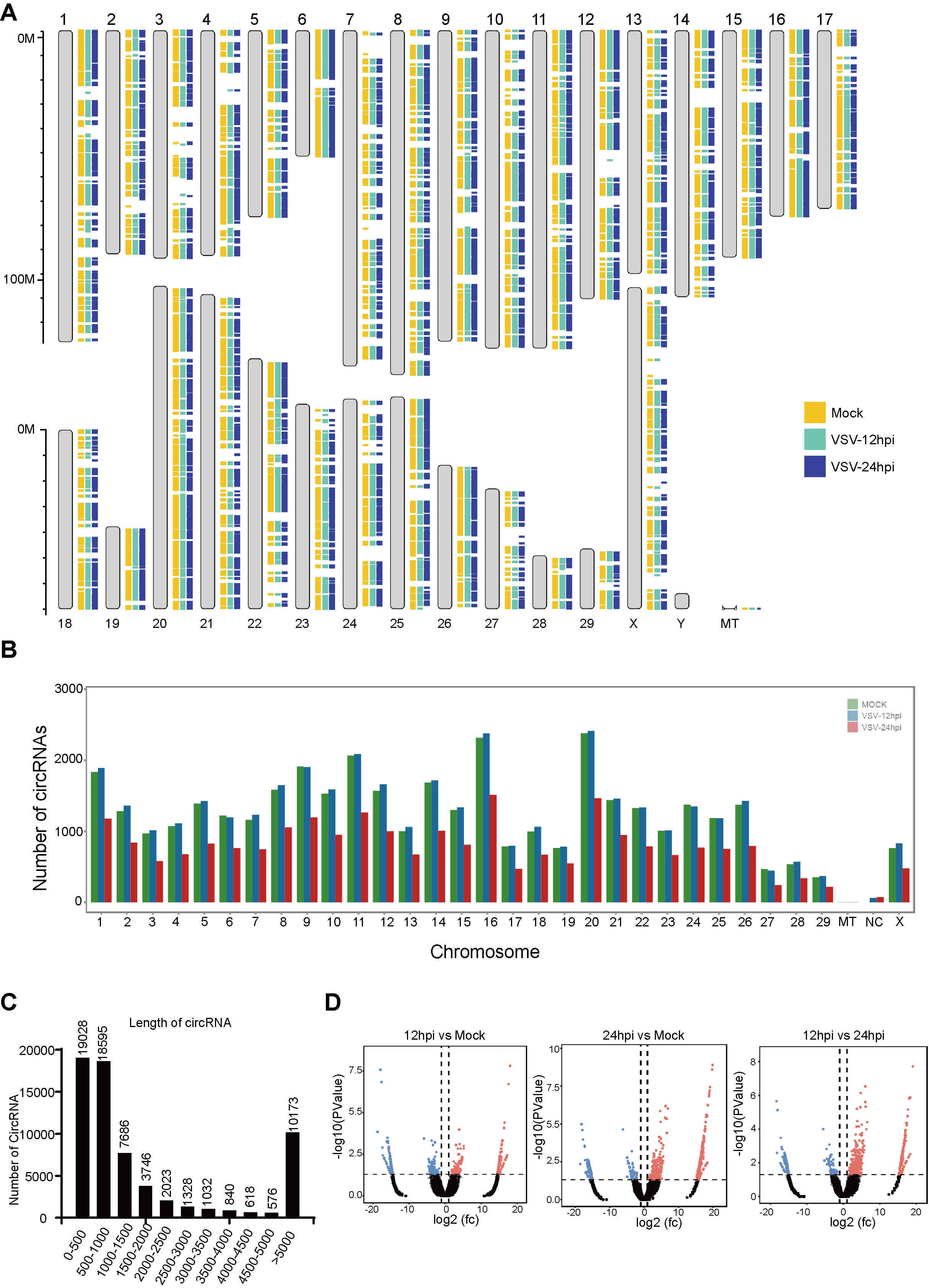

Supplement: Supplementary file 1 [file Image1.tif]

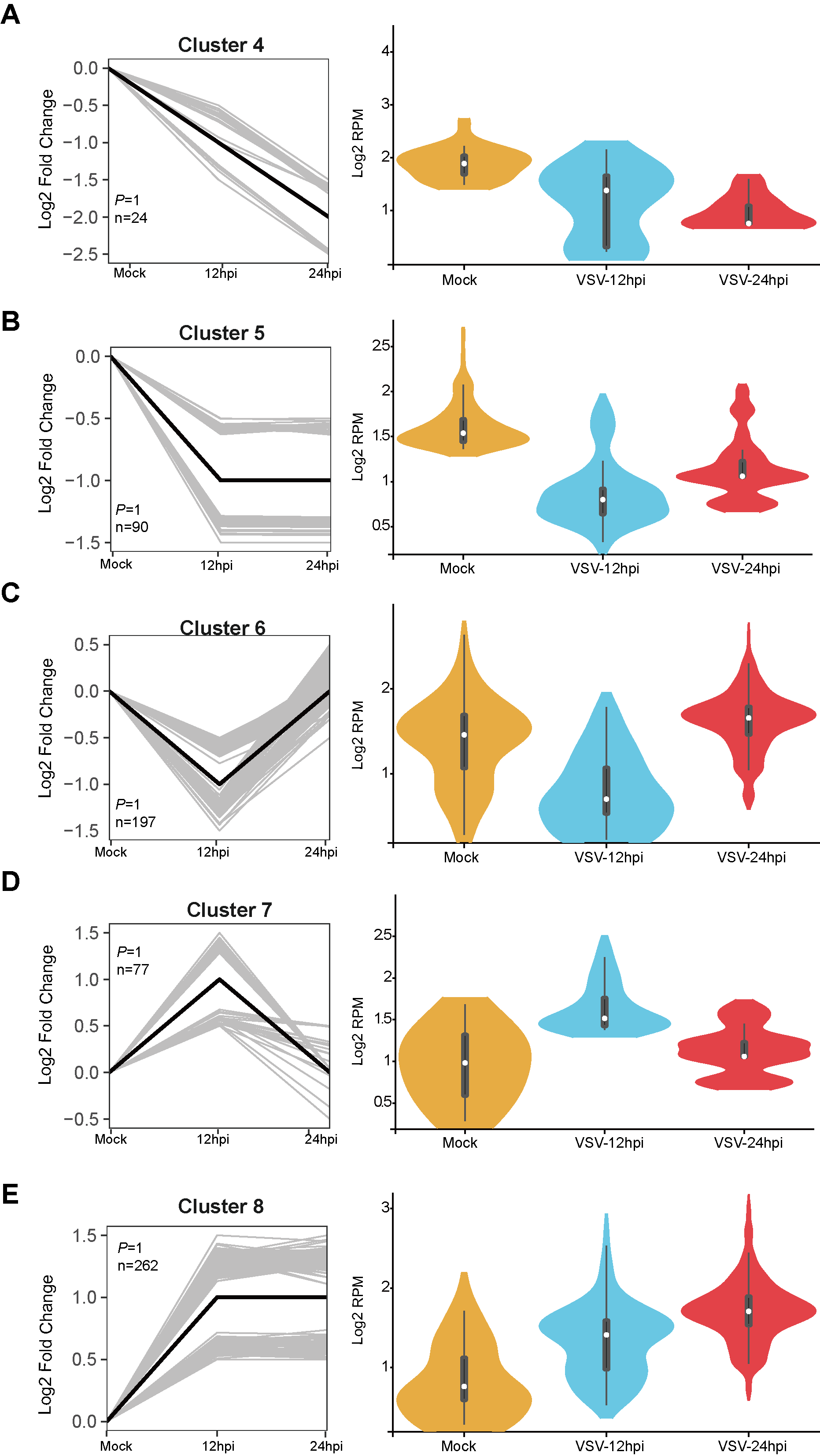

Supplement: Supplementary file 2 [file Image2.tif]

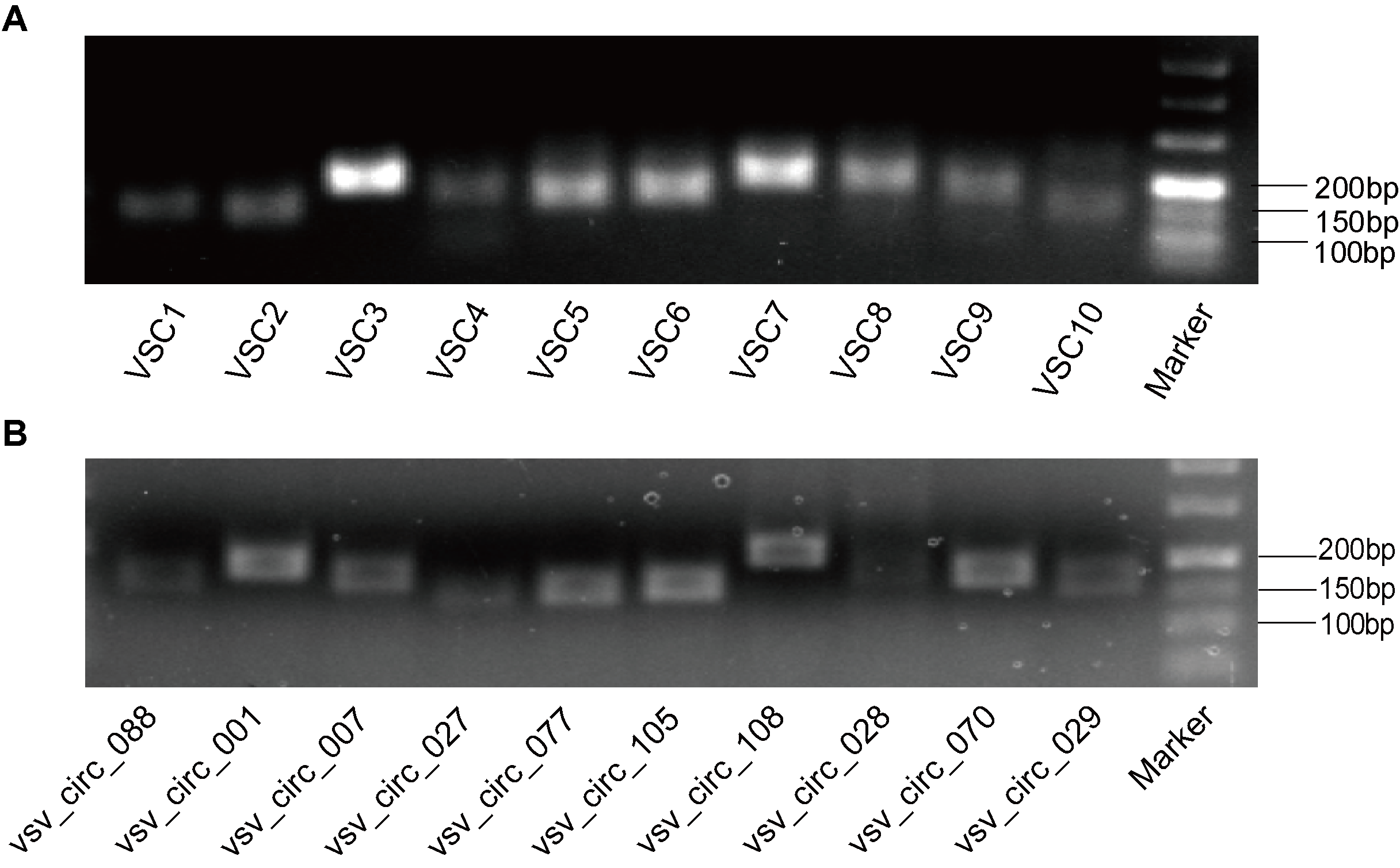

Supplement: Supplementary file 3 [file Image3.tif]

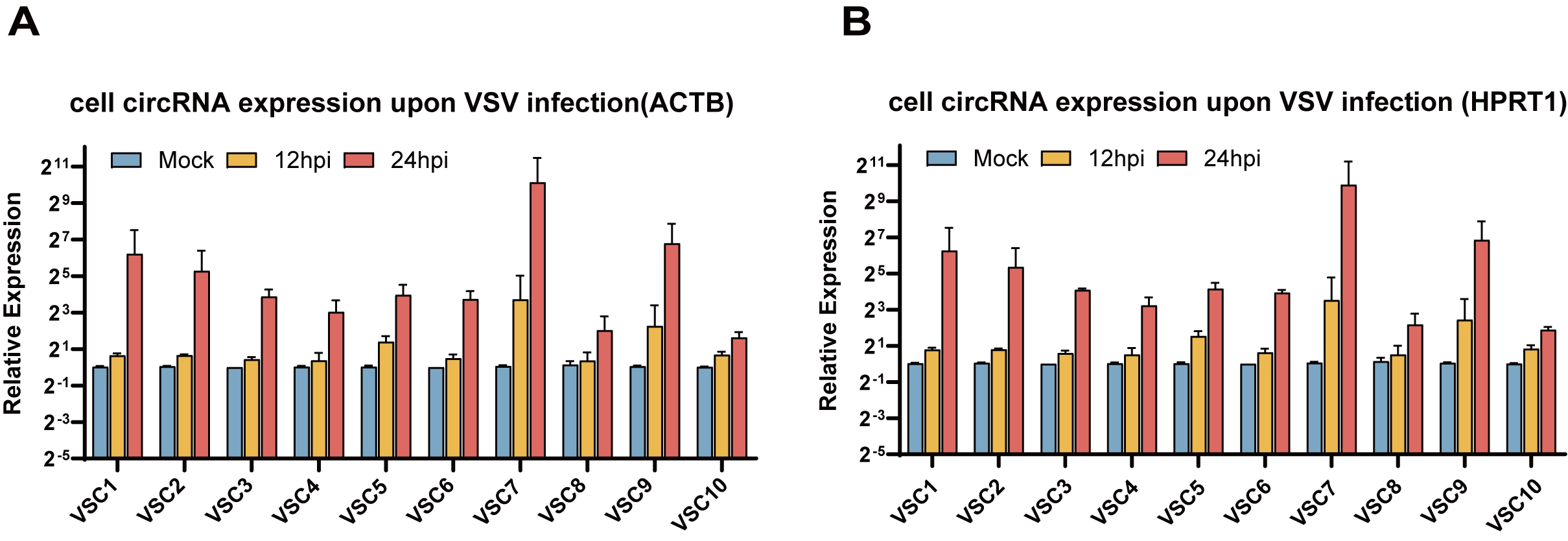

Supplement: Supplementary file 4 [file Image4.tif]
