# Supplementary material for: Comprehensive profiling of host- and virus-derived circular RNAs during vesicular stomatitis virus infection
Source: Front Cell Infect Microbiol. 2025 Oct 16;15:1654185. doi: 10.3389/fcimb.2025.1654185 (PMC12571801; doi:10.3389/fcimb.2025.1654185)
Supplement: Supplementary file 5 [file Table1.docx]

**Supplementary Table 1** Primer used in this study

| **Alias Name** | **Sequences (5'--> 3')** |
| --- | --- |
| VSC1 | F: agaggaccaagctctgatgag; R: tgaaactggttcacaggcttg |
| VSC2 | F: tacacactcaagacggtcac; R: cacgtcctggccctccattg |
| VSC3 | F: acgtggtgcagaatatccag; R: cctctcagcagacgctgaag |
| VSC4 | F: ctctgaccatcaccgtcttc; R: ctcatcttgcctgggctcag |
| VSC5 | F: ccaaccttcccgtgcacatc; R: acactgcccaggacactctc |
| VSC6 | F: attcagacaaacaaagcttc; R: ggctgctggttacaaatctg |
| VSC7 | F: attgaacttcccagcactcg; R: acttacctctggggtgttag |
| VSC8 | F: caagacctgaaggatgggac; R: gatgtagaagaggagtccac |
| VSC9 | F: tgattgtggcagaagatgtc; R: tgagggaaggtaagtcatag |
| VSC10 | F: tctactgcaaagagcctatc; R: atagcactcactgcagttac |
| vsv_circ_001 | F: aggcatctctagcatctttg; R: aagaagaccggtctatactg |
| vsv_circ_007 | F: tatggtatgtaaaggcttggg; R: atgttccataagtcttgtag |
| vsv_circ_027 | F: tggattgtcttctaagtctc; R: atcctcttagattcaatgtc |
| vsv_circ_028 | F: taggtgtccatctcgtctgag; R: gaaactggaggcaaggcctg |
| vsv_circ_029 | F: caatataggtctttacaagg; R: gaatttctcatataatcagg |
| vsv_circ_070 | F: ctgtctctgagatgactatg; R: agttcttactatcccacatc |
| vsv_circ_077 | F: ataaacatcgggaaagcagg; R: cattttgattaaaaggcaac |
| vsv_circ_088 | F: ttgattaaaatggtggaattg; R: atcaagtttcttgaagtaagc |
| vsv_circ_105 | F: ggcagagatgtggtcgaatg; R: caatttcttggcttagcatac |
| vsv_circ_108 | F: gcatctctaagacgtgtagc; R: cctccattcctttctcttac |
